# Changes in the outcome of prostate biopsies after preventive task force recommendation against prostate-specific antigen screening

**DOI:** 10.1186/s12894-018-0384-x

**Published:** 2018-08-20

**Authors:** Ahmed S. Zakaria, Alice Dragomir, Fadi Brimo, Wassim Kassouf, Simon Tanguay, Armen Aprikian

**Affiliations:** 1 Department of Surgery, Division of Urology, McGill University, McGill University Health Centre, 1001 Boulevard Decarie, Montreal, Quebec, H4A 3J1 Canada; 2 Department of Pathology, McGill University, Montreal, Quebec, Canada

**Keywords:** Prostate-specific antigen, Prostate cancer screening, Task force recommendations, Prostate biopsies

## Abstract

**Background:**

The benefits of PSA-based screening for prostate cancer (PCa) are controversial. The Canadian and American Task Forces on Preventive Health Care (CTFPHC & USPSTF) have released recommendations against the use of routine PSA-based screening for any men. We thought to assess the impact of these recommendations on the outcomes and trends of prostate needle biopsies.

**Methods:**

A complete chart review was conducted for all men who received prostate needle biopsies at McGill University Health Center between 2010 and 2016. Of those, we included 1425 patients diagnosed with PCa for analysis. We Compared 2 groups of patients (pre and post recommendations’ release date) using Welch’s t-tests and Chi-square test. A multivariate logistic regression model was used to analyze variables predicting worse pathological outcomes.

**Results:**

When the release date of the USPSTF draft (October 2011) was used as a cut-off, we found an average annual decrease of 10.6% in the total number of biopsies. The median (IQR) baseline PSA levels were higher in post-recommendations group (*n* = 977) when compared to pre-recommendations group (*n* = 448) [8 ng/ml (5.7–12.9) versus 6.4 ng/ml (4.9–10.1), respectively. *P* = 0.0007]. Also, post-recommendations group’s patients had higher Gleason score (G7: 35.4% versus 28.4% and G8-G10: 31.2% versus 18.1%, respectively. *P* < 0.0001). Moreover, they had higher intermediate and high-risk PCa classification (36.4% versus 32.8% and 35.5% versus 22.1%, respectively. *P* < 0.0001). The recommendations release date was an independent variable associated with higher Gleason score in prostate biopsies (OR: 2.006, 95%CI: 1.477–2.725). Using the CTFPHC recommendations release date (October 2014) as a cut-off in further analysis, revealed similar results.

**Conclusions:**

Our results revealed a reduction in the number of prostate needle biopsies performed over time after the recommendations of the preventive task forces. Furthermore, it showed a significant relative increase in the higher risk PCa diagnosis. The oncological outcomes associated with this trend need to be examined in further studies.

## Background

Prostate cancer (PCa) is the most frequently diagnosed cancer in men, with an estimated 202,490 new cases diagnosed in North America in 2016 [1, 2]. In Canada, it is expected that 1 in 8 males will develop PCa in their lifetime and last year it accounted for 10% of cancer-related death in Canadian men [2].

Owing to the high incidence rate and the potential for cure with early detection, screening for PCa using the prostate specific antigen (PSA) blood test, is a common practice. Since its emergence in 1986 [3] and its approval by the Food and Drug Administration in 1994 [4], along with the digital rectal exam (DRE), PSA has been shown to be a valuable oncological marker. Epidemiologically this approach was associated with a dramatic increase in PCa detection rates and substantial decline in PCa mortality rates that have fallen by over 50% [5].

However during the last decade, results from major randomized trials, showed mixed evidence regarding the utility of PSA screening, with questionable survival benefit and significant harms associated with PCa diagnosis and overtreatment [6–8]. Following these results, the US Preventive Services Task Force (USPSTF) issued a first recommendation in 2008 advising against routine screening in men older than 75 years [9]. Few years later in October 2011 they issued the highly publicized draft recommendation against PSA screening of all ages, that was finalized as (Grade D recommendation) in May 2012 [10]. Recently, in October 2014 the Canadian Task Force on Preventive Health Care (CTFPHC) issued a similar recommendation against PCa screening with PSA.

The USPSTF and CTFPHC recommendations may have changed screening practice and referral patterns among primary care physicians [11–14]. In the current study, we aimed to characterize the trends of prostate needle biopsies as well as to assess for changes in the pathological outcomes before and after these recommendations, in a tertiary-care academic hospital.

## Methods

### Data source and study population

The study cohort was built retrospectively through complete chart review, during the period between January 2010 and December 2016, to analyze data of all patients who underwent prostate biopsies at McGill University Health Center. Patients’ information was collected in a database with an institutional review board-approved protocol for the collection of data. Our cohort’s patients were referred to our tertiary-hospital by primary care providers and were offered trans-rectal ultrasound (TRUS)-guided prostate biopsy to rule out PCa due to abnormal laboratory or clinical findings. Data collected included: demographics, laboratory, clinical, and pathological data in relation to the first recorded prostate needle biopsy. From our whole cohort, the exclusion criteria of this study were: 1) patients who were previously diagnosed with PCa; 2) repeated biopsies of active surveillance patients; 3) absent baseline PSA test result; and 4) non-standard needle biopsies (non-TRUS-guided prostate biopsy and biopsies with less than 10 cores).

### Biochemical, clinical and pathological evaluation

The baseline serum PSA level was defined as the last PSA measured before the diagnostic biopsy and up to 3 months before biopsy. PSA was categorized based on D’Amico risk score criteria as patients with a PSA of 10.00 ng/ml or less (sub-categorized into: 4.00 ng/ml or less and 4.01 to 10.00 ng/ml), 10.01 to 20.00 ng/ml and greater than 20.00 ng/ml. Patient’s PSA density (PSAD) was calculated by dividing baseline PSA to prostate volume measured by TRUS at time of diagnostic biopsy. Clinical staging was determined from the TRUS findings at the time of diagnostic biopsy or by digital rectal exam (DRE) at the time of first encounter with the urologist. Individual D’Amico risk classification score was calculated for each patient using previously published criteria [15].

Prostate needle biopsies were performed during the study period by six attending urologists and radiologists, with the number of cores taken per biopsy varying according to the time period of the biopsy (range, 10–20 cores). All prostate biopsies specimens were reviewed by a team of four attending pathologists led by dedicated genitourinary pathologist (F.B.). The biopsy findings analyzed in this study included: the Gleason score (primary and secondary predominant patterns), the total number of cores, the number of positive cores, and the maximum percentage of cancer on each core. The modifications of the Gleason grading system, implemented by the International Society of Urological Pathology over the previous years, were taken in account during reporting.

### Statistical analysis

Descriptive statistics [percentages for categorical variables and mean (standard deviation = SD or range) and medians (inter quartile range = IQR) for continuous variables], respectively, were used to summarize the characteristics of the study population. Age, age categories, baseline PSA, PSA categories, baseline prostate volume, PSA density, Gleason score on biopsy, clinical stage and D’Amico risk classification were compared between patients who underwent prostate needle biopsies before recommendation date (USPSTF draft on 7 October 2011 and CTFPHC recommendations on 27 October 2014) with those who underwent prostate needle biopsies after the recommendation release date. Comparison between groups was performed using Chi-square and Welch’s t-tests. Multivariate logistic regression models were used to assess the association between the time period of prostate biopsy (pre- versus post-recommendation date) and worse pathological outcomes while adjusting for potential confounding factors and other covariables. Analyses were performed using the Statistical Analysis System Software (Version 9 SAS Institute, Cary, North Carolina). All tests were two-sided with a significance threshold of 5%.

## Results

**Fig. 1:**
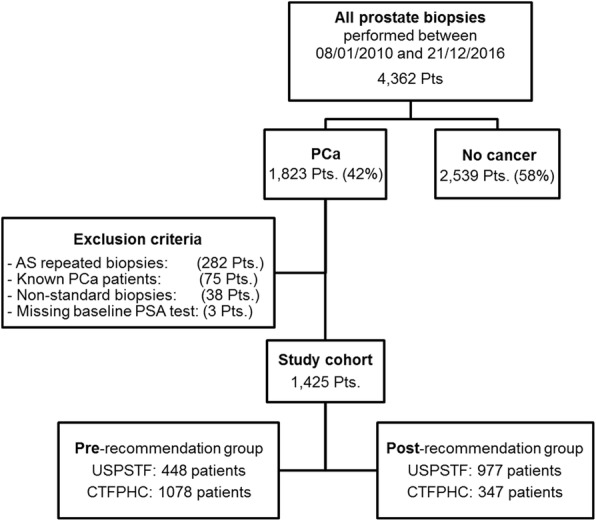
Study flowchart and exclusion criteria

Our study flowchart is shown in Fig. 1. From our main cohort of all patients (4362 patients) who underwent prostate biopsies between January 2010 and December 2016, 1823 (41.7%) patients were diagnosed with PCa and finally 1425 (32.6%) patients were included for analysis after applying the study’s exclusion criteria (excluded 298 (9.1%) patients).

**Fig. 2:**
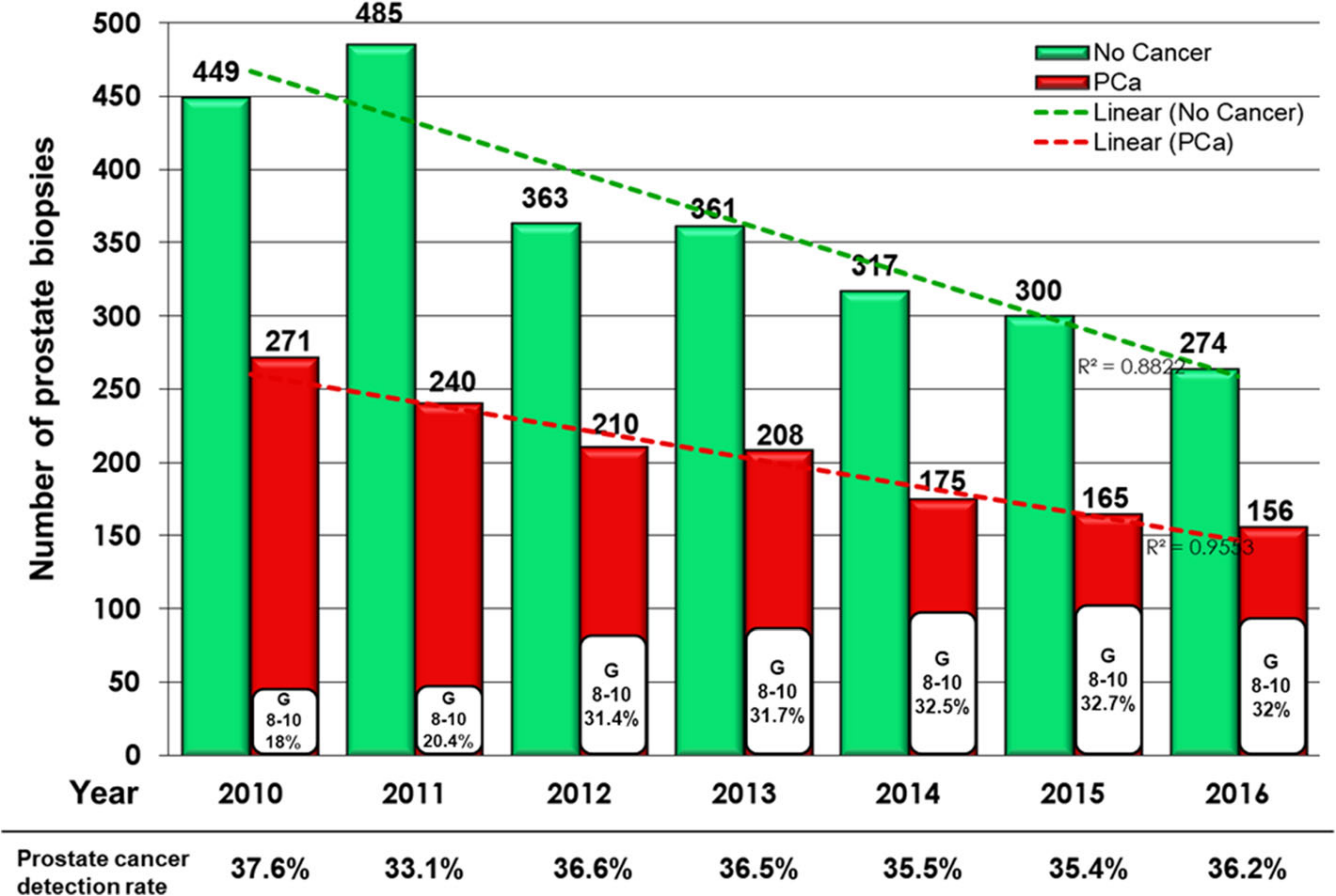
Rate of prostate needle biopsies performed over study period (2010–2016), with absolute numbers of cases negative for PCa (represented by green columns) and absolute numbers of cases positive for PCa (represented by red columns), percentage of cases diagnosed with Gleason grades G8–10 were calculated to the year positive cases (represented by white columns)

During the study time period, there was a trend of decline in the total number of prostate needle biopsies performed over years as shown in Fig. 2, with an average annual decrease of 10.6% after the recommendations, this trend was consistent for biopsies pathologically diagnosed as PCa and biopsies that showed no evidence of cancer. Also, our results revealed that relative PCa detection rate did not change significantly over study period (*p = 0.24*), specially before and after the (USPSTF & CTFPHC) recommendations, but what actually has changed was the percentage of pathologically high grade cancer diagnosed, namely G8–10, that increased by at least 11% in the years immediately after the release of the 2011 USPSTF draft recommendations.

The baseline clinical, biochemical and pathological characteristics of the study population were noted in Table 1. Basically, at the time of diagnosis, the cohort mean (SD) age was 68.6 (8.72) years, The baseline PSA was ≤10 ng/mL in 948 (66.5%) patients, 566 (39.7%) patients had a Gleason score of ≤6 and 477 (33.4%) patients were classified as having low risk PCa according to D’Amico risk classification criteria.

**Table 1:** Baseline clinical, biochemical and pathological features of the cohort

Parameters	Value
Clinical:	
Cohort total number of patients:	1425
Age at diagnosis, years, mean (SD):	68.6 (8.72)
Age categories, n (%)	
- < 50 years	8 (0.6%)
- 50–74 years	861 (60.4%)
- ≥75 years	556 (39%)
Baseline prostate volume (ml^3^), median [IQR]:	36.4 [27–49.8]
Clinical stage: n (%)	
- T1-2a	859 (60.3%)
- T2b	355 (24.9%)
- T2c-3a	211 (14.8%)
Biochemical:	
Baseline PSA (ng/ml), median [IQR]	7.55 [5.4–12]
PSA categories, ng/ml, n (%)	
- ≤ 4:	113 (7.9%)
- 4.1–10:	835 (58.6%)
- 10.1–20:	293 (20.6%)
- > 20	184 (12.9%)
PSA density (ng/ml/ml^3^), median [IQR]	0.21 [0.13–0.36]
Pathological:	
Gleason score, n (%)	
- Gleason ≤6:	566 (39.7%)
- Gleason 7:	473 (33.2%)
- Gleason 8:	194 (13.6%)
- Gleason 9:	168 (11.8%)
- Gleason 10:	24 (1.7%)
D’Amico risk classification, n (%)	
- Low:	477 (33.4%)
- Intermediate:	503 (35.3%)
- High	445 (31.2%)

*SD* Standard Deviation, *IQR* Inter Quartile Range, *n (%)*: Number of patients (Percentage)

The absolute numbers of different pathological grades diagnosed in biopsies during the study time period are shown in Table 2, where there was an increase in the number of G8–10 cases after the USPSTF recommendation, associated with decline in low grade cancer, especially G6 cases during the same time period.

**Table 2:** Absolute numbers of Gleason grades diagnosed during the study time period

	Year						
Grade	2010	2011	2012	2013	2014	2015	2016
G8–10	49	49	66	66	57	54	50
G7	77	71	55	75	68	75	59
G6	145	120	89	67	50	36	47
Total	271	240	210	208	175	165	156

Based on USPSTF draft recommendation as a cut-off, Table 3 is comparing characteristics of 448 patients in the pre-recommendation group with 977 patients in the post-recommendation group. Generally, patients in the post-recommendation group were relatively younger, and had significantly higher median baseline PSA of 8 ng/ml compared to a median baseline PSA of 6.4 ng/ml in the pre-recommendation group (*p* = 0.0007). Moreover, 37.4% of the post-recommendation group had a PSA more than 10 ng/ml at diagnosis in comparison to 25.2% in the pre-recommendation group (*p* < .0001). With respect to the clinical and pathological criteria, patients in the post-recommendation group were more likely to have higher clinical stage, where 171 (17.5%) patients had T2c-3a compared to 40 (9%) patients with the same stage in the pre-recommendation group, as well as higher Gleason score at diagnostic biopsies, where 305 (31.2%) patients in the post-recommendation group had Gleason grades 8–10 in comparison to 81 (18.1%) patients in the pre-recommendation group (*p* < .0001). Post recommendation patients were more likely to be classified into the D’Amico high risk PCa category (35.5% versus 22.1%, *p* < .0001).

**Table 3:** Comparison between groups (according to USPSTF recommendation)

Characteristics	Pre-recommendation 448 Pts. (31.5%)	Post-recommendation 977 Pts. (68.5%)	*P*-value
Age, median [IQR]	74 [68–80]	71 [65–77]	
Age at diagnosis, mean [range]	68.2 [41–96]	68.8 [40–93]	0.258
Age categories, n (%)			<.0001
- < 50 years	1 (0.2%)	7 (0.7%)	
- 50–74 years	234 (52.2%)	627 (64.2%)	
- ≥75 years	213 (47.6%)	343 (35.1%)	
PSA (ng/ml), median [IQR]	6.4 [4.9–10.1]	8 [5.7–12.9]	0.0007
PSA categories, n (%)			<.0001
- ≤4	48 (10.7%)	65 (6.6%)	
- 4.01–10	287 (64.1%)	548 (56.1%)	
- 10.01–20	76 (16.9%)	217 (22.2%)	
- > 20	37 (8.3%)	147 (15.1%)	
Prostate volume (ml3), median [IQR]	34.9 [26–46.2]	37.2 [27.1–51]	0.126
PSA density (ng/ml/ml3), median [IQR]	0.19 [0.13–0.33]	0.22 [0.14–0.39]	0.132
Gleason score			<.0001
- Gleason 6	240 (53.5%)	326 (33.4%)	
- Gleason 7	127 (28.4%)	346 (35.4%)	
- Gleason 8–10	81 (18.1%)	305 (31.2%)	
Clinical stage			<. 0001
- T1-2a	301 (67.1%)	558 (57.1%)	
- T2b	107 (23.9%)	248 (25.4%)	
- T2c-3a	40 (9%)	171 (17.5%)	
D’Amico risk classification			<. 0001
Low	202 (45.1%)	275 (28.1%)	
Intermediate	147 (32.8%)	356 (36.4%)	
High	99 (22.1%)	346 (35.5%)	

Applying the Canadian Task force recommendation date and dividing the cohort similarly into two groups, before and after the recommendation date yielded similar results as shown on Table 4.

**Table 4:** Comparison between groups (according to CTFPHC recommendation)

Characteristics	Pre-recommendation 1078 Pts. (75.6%)	Post-recommendation 347 Pts. (24.3%)	*P*-value
Age, median [IQR]	73 [67–79]	69 [64–76]	
Age at diagnosis, mean [range]	68.4 [40–96]	69.1 [47–91]	0.274
Age categories, n (%)			<.0001
- < 50 years	4 (0.4%)	4 (1.1%)	
- 50–74 years	615 (57%)	246 (70.8%)	
- ≥75 years	459 (42.6%)	97 (27.9%)	
PSA (ng/ml), median [IQR]	7.1 [5.2–11.5]	8.8 [6.4–13.4]	0.037
PSA categories, n (%)			0.011
- ≤4	90 (8.4%)	23 (6.6%)	
- 4.01–10	652 (60.4%)	183 (52.7%)	
- 10.01–20	204 (18.9%)	89 (25.7%)	
- > 20	132 (12.2%)	52 (15%)	
Prostate volume (ml3), median [IQR]	34.8 [26.4–47.1]	40.3 [29.6–56.9]	<.0001
PSA density (ng/ml/ml3), median [IQR]	0.21 [0.14–0.336	0.22 [0.12–0.38]	0.756
Gleason score			<.0001
- Gleason 6	473 (43.9%)	93 (26.8%)	
- Gleason 7	327 (30.3%)	146 (42.1%)	
- Gleason 8–10	278 (25.8%)	108 (31.1%)	
Clinical stage			0.035
- T1-2a	669 (62.1%)	190 (54.7%)	
- 2b	252 (23.4%)	103 (29.7%)	
- 2c-3a	157 (14.5%)	54 (15.6%)	
D’Amico risk classification			<.0001
- Low	399 (37%)	78 (22.4%)	
- Intermediate	356 (33%)	147 (42.4%)	
- High	323 (30%)	122 (34.8%)	

Results from the multivariate logistic analyses for the variables predicting worse pathological outcomes in the prostate needle biopsies, showed that the US task force recommendation release date was an independent variable associated with higher Gleason score (G8–10) in biopsies, with patients who had their biopsies performed after the recommendation release date having double the odds of being diagnosed with Gleason score 8–10 (OR: 2.006, 95%CI: 1.477–2.725) as illustrated in Table 5.

**Table 5:** multivariate analyses of factors predicting higher Gleason score on biopsies

Variable	Odds ratio estimates		
	Point estimate	95% Confidence interval	*P*-Value
Post USPSTF recommendation	2.006	1.477–2.725	<.0001
Post CTFPHC recommendation	1.359	0.980–1.868	0.058
Age at diagnosis	1.047	1.032–1.063	<.0001
Baseline PSA	1.074	1.032–1.063	<.0001
Baseline prostate volume	0.990	0.983–0.997	0.006
Baseline PSA density	1.181	0.596–2.343	0.633
Number of cores per biopsy	1.035	0.953–1.124	0.409
TRUS operator (Urologist versus Radiologist)	1.326	0.989–1.778	0.060
Pathology reviewer (F.B. versus others)	1.013	0.793–1.294	0.916

## Discussion

Over the past few decades, the introduction of the serum PSA test has been associated with a greater than 50% significant reduction in PCa mortality rates in many areas around the world [5]. It is believed that this downward mortality path is attributed mainly to the PSA-based screening programs and improved treatment strategies. However, despite this decline in PCa specific mortality rates since the early 1990s, controversy about the harms and benefits of PSA based screening still exist [16].

The harms of PSA screening are well known including overdiagnosis and overtreatment. The USPSTF and CTFPHC recommendations against PSA screening were based mainly on three significant randomized controlled trials, namely, the Prostate, Lung, Colon and Ovarian (PLCO) screening trial, the European Randomized Study of Screening for Prostate Cancer (ERSPC) and the Goteborg trial [6, 7, 17].

The task forces have a significant effect on the practice patterns of primary and specialty healthcare professionals, as seen with primary care providers with whom the decision to offer screening usually lies [18].

Following the draft guidelines in October 2011 and the official recommendations against PSA screening in May 2012 in US and October 2014 in Canada, multiple studies demonstrated a significant decrease in PSA screening. Shoag et al. [11] used the US National Ambulatory Medical Care Survey (NAMCS) data and recently reported a relative 64% decrease in DRE and a 39% decrease in PSA testing after the recommendations. The decrease was significant among men 55 to 69 years old, where the number of visits in which DRE and PSA testing were performed decreased 65% and 39%, respectively (*p* < 0.001).

Drazer et al. [12] reported a significant decline in PSA-based screening after the recommendations, using the US National Health Interview Survey (NHIS) data, with the largest decline among men aged 50–59 years, where relative screening rates decreased by 25% from 2010 to 2013. Similarly, Jemal et al. [13] showed a decrease in the PSA screening rates by 18% between 2010 and 2013 and as in the previous reports, the highest decline was seen among men aged 50–74 years.

On the other hand, Hutchinson et al. [19] did not identify a significant change in the use of PSA-based screening as measured by the total annual number of resultant PSA examinations in their single-center analysis. However, they reported that patients were referred at progressively higher average PSA levels. Also, Rahbar et al. [20] in their recent update of their already published data (14) extended the previous data analysis with additional years to determine if the downward trend continued past the immediate response to the recommendation, and they showed that from 2013 to 2015 there was a non-significant decrease in PSA screening (only 0.4%). According to them, the absence of a change between these years might highlight the contrasting recommendations by different guideline panels regarding the use of this test.

In our study we present the finding of a significant decline in prostate biopsy volume following the USPSTF and CTFPHC recommendations, where we found an average annual decrease of 10.6%. Our results matched a recent report from a community-based urology practice, where Gaylis et al. [21] examined a total of 3915 prostate biopsies performed during 4 years, with 1581 (40.4%) of these prostate biopsies performed in men referred for newly elevated PSA. They found a 22.8% reduction in biopsies performed in newly referred men. Also in Canada, Bhindi et al. [22] conducted a time series analysis during 2008 to 2013 of prostate biopsies performed at University Health Network in Toronto, and reported a decline in the median number of biopsies performed per month from 58.0 (IQR 54.5–63.0) before the USPSTF recommendations to 35.5 (IQR 27.0–41.0) afterward (*p* = 0.003). Likewise, Banerji et al. [23] assessed the number of needle biopsies done at an academic institution in the US during the 30-month period before and after the USPSTF recommendation and reported a 31% decrease in the absolute number of biopsies. Furthermore, Gershman et al. [24], showed that prostate biopsy rates dropped by 33% from 64.1 to 42.8 per 100,000 person/months from 2005 to 2014, with the greatest decrease following the 2012 USPSTF recommendation (− 13.8; 95% CI, − 21.0 to − 6.7; *p* < 0.001).

Halpern et al. [25] conducted a US national study across academic and community practice settings and health plans to evaluate variations in prostate biopsy volumes from 2009 through 2015, they demonstrated geographic variation in prostate biopsy volumes and an overall decrease in prostate biopsies after USPSTF recommendation, the median biopsy volume per urologist significantly decreased from 29 to 21 (IQR 12–34; *p* < 0.001), and the total number of annual biopsies decreased by 12.7%. After adjustment for practice and physician characteristics, they reported an overall decrease of 28.7% in biopsy volume following 2012. The greatest decrease in biopsy volume was observed in men with abnormal PSA, whereas biopsy volume in men under surveillance for confirmed PCa significantly increased by 28.8%.

Conversely, Misra-Hebert et al. [26] in their study conducted over 160,211 men aged ≥40 years with at least one visit to a primary care clinic during the years 2007–2014, reported higher rates of first prostate biopsy in men who were screened with a PSA test, especially for men with an increased risk of PCa (African Americans and men with positive family history). However, when they used all men aged ≥40 years with a primary care clinic visit each year as the denominator, overall yearly rates of prostate biopsy were similar between 2007 and 2014 and for men ≥70 years, biopsy rates decreased in 2014 in comparison to 2007.

In our study, PSA assessments over time revealed that for men presenting for prostate biopsy, the median PSA values showed a rising trend after recommendations. This trend was significant for both USPSTF and CTFPHC recommendations (*p* = 0.0007 and 0.037, respectively). In addition the percentage of men presenting with PSA value > 10 ng/ml was significantly higher in the post recommendation era (*p* < .0001 and 0.011, respectively). These findings are consistent with previous two studies [21, 23], where one reported that post-USPSTF patients had a higher median PSA (*p* < 0.001), and was significantly more likely to have a PSA between 6.1 and 10 ng/ml (*P* = 0.019) or 10.1 and 20 ng/ml (*p =* 0.002) than the pre-USPSTF patients. The second study reported that the proportion of men presenting with PSA > 10 ng/ml increased from 28.1 to 36.8% (*p* = 0.009).

Among our cohort, we found no significant changes in the relative PCa detection rate (33.1% to 37.6%) over the study period. However, we noted worse pathological outcomes in terms of slight higher absolute numbers and rates of Gleason grades [8–10] and higher risk classification PCa cases diagnosed in the years after the recommendations. Similarly, Hu et al. [27] Using the most recent Surveillance, Epidemiology, and End Results (SEER) release, identified 1,107,111 men 40 years or older diagnosed with PCa from 2004 to 2013 and reported increase in the percentage presenting with intermediate and high-grade PCa, from 46.3 to 56.4% (*p* < .01), in men younger than 75 years, and increase in the proportion of men presenting with distant metastases from 2.7 to 4.0% (*p* < .01). Bhindi et al. [22] in their study also reported no significant differences in relative cancer detection rates in the year after versus the year before USPSTF recommendations, but In contrast with our results, they found significant decrease (*p* < 0.001) in the absolute rates of cancer detection after the USPSTF recommendation statement, where the median number of Gleason 7–10 PCa detected per month decreased from 17.5 (IQR 14.5–21.5) to 10.0 (IQR 9.0–12.0), however, their report was limited to only one year after the recommendation.

Although our results showed higher rates of high-risk PCa after the recommendation, the actual absolute number was a little higher, which may be explained by the pattern of less aggressive screening during the years after the recommendations, which led to decreased absolute numbers and rates of low grade PCa detected during screening.

Barocas et al. [28] investigated the incident diagnoses of PCa after the USPSTF draft recommendation, based on US national cancer database, they reported 28% decrease in the incidence, they noted that the monthly PCa diagnoses decreased by 1363 cases (12.2%, *p* < 0.01) in the month after the USPSTF draft and continued to decrease by 164 cases per month relative to baseline (− 1.8%, *p* < 0.01). Jemal et al. [13] reported more specific decreases in the early-stage PCa incidence following the 2012 USPSTF recommendations. The largest decrease occurred between 2011 and 2012, from 498.3 to 416.2 per 100,000 men aged 50 years and older. In addition they recently updated their results [29] and reported a continuing decline in incidence rates for early-stage PCa in men aged over 50 years, the decrease rate was lower in 2012–2013 than that from 2011 to 2012 (6% versus 19%).

Of note, recently the US task force initiated a new update process of the 2012 recommendation on PCa screening and in April 2017 they issued a new draft recommendation, that was published as final recommendation as of May 2018 [30], proposing the following modification based on additional evidence published since the 2012 recommendation: - For men aged 55–69: The decision about whether to be screened for PCa should be an individual one. The USPSTF recommends that clinicians inform men ages 55 to 69 years about the potential benefits and harms of PSA–based screening for PCa. (Grade C).

Our study has some limitations including its retrospective nature, single center experience (related to local network of primary care physician), and being an observational study that cannot confirm causality. Despite these limitations, the strengths of our study include being the first study to assess and report on prostate biopsy outcomes after both (the US and Canadian) recommendations, the fair number of patients included and the longer follow up time after the recommendations. We believe that our study results with the results of others could be informative to the health policy makers.

## Conclusions

In conclusion, our results revealed a reduction in the total number of prostate needle biopsies performed over time after the recommendations of the American and Canadian preventive task forces against PSA-based screening for prostate cancer. Furthermore, it showed a slight increase in absolute high-risk PCa diagnoses and a significant relative increase in higher risk PCa diagnosis. The oncological outcomes associated with this trend need to be examined in further studies.

## Abbreviations

CTFPHC: Canadian Task Force on Preventive Health Care
DRE: Digital rectal exam
IQR: Inter Quartile Range
PCa: Prostate Cancer
PSA: Prostate Specific Antigen
PSAD: Prostate Specific Antigen Density
SD: Standard Deviation
TRUS: Trans-Rectal Ultrasound
USPSTF: US Preventive Services Task Force
